# Mutational analysis of multiple lung cancers: Discrimination between primary and metastatic lung cancers by genomic profile

**DOI:** 10.18632/oncotarget.16096

**Published:** 2017-03-10

**Authors:** Taichiro Goto, Yosuke Hirotsu, Hitoshi Mochizuki, Takahiro Nakagomi, Daichi Shikata, Yujiro Yokoyama, Toshio Oyama, Kenji Amemiya, Kenichiro Okimoto, Masao Omata

**Affiliations:** ^1^ Lung Cancer and Respiratory Disease Center, Yamanashi Central Hospital, Yamanashi, Japan; ^2^ Genome Analysis Center, Yamanashi Central Hospital, Yamanashi, Japan; ^3^ Department of Pathology, Yamanashi Central Hospital, Yamanashi, Japan; ^4^ University of Tokyo, Tokyo, Japan

**Keywords:** lung cancer, multiple cancers, metastasis, mutation, next-generation sequencing

## Abstract

In cases of multiple lung cancers, individual tumors may represent either a primary lung cancer or both primary and metastatic lung cancers. Treatment selection varies depending on such features, and this discrimination is critically important in predicting prognosis. The present study was undertaken to determine the efficacy and validity of mutation analysis as a means of determining whether multiple lung cancers are primary or metastatic in nature. The study involved 12 patients who underwent surgery in our department for multiple lung cancers between July 2014 and March 2016. Tumor cells were collected from formalin-fixed paraffin-embedded tissues of the primary lesions by using laser capture microdissection, and targeted sequencing of 53 lung cancer-related genes was performed. In surgically treated patients with multiple lung cancers, the driver mutation profile differed among the individual tumors. Meanwhile, in a case of a solitary lung tumor that appeared after surgery for double primary lung cancers, gene mutation analysis using a bronchoscopic biopsy sample revealed a gene mutation profile consistent with the surgically resected specimen, thus demonstrating that the tumor in this case was metastatic. In cases of multiple lung cancers, the comparison of driver mutation profiles clarifies the clonal origin of the tumors and enables discrimination between primary and metastatic tumors.

## INTRODUCTION

In cases of synchronous or metachronous multiple cancers, individual tumors may be comprised of either a primary lung cancer or both primary and metastatic lung cancers. Treatment selection is dependent on such features. Usually, in cases of multiple lung cancers, whether a tumor is metastatic or primary can be judged on the basis of its clinical course, diagnostic imaging findings and/or pathology. In particular, when diagnostic imaging findings are assessed, a tumor is suspected of being metastatic if multiple small nodules appear simultaneously and assume a round and smooth-surfaced form. In that case, primary lung cancer is usually large, while its metastatic sites within the lung are often smaller in comparison. Thus, the coexistence of a large tumor with multiple smaller nodules within the lung strongly suggests metastasis. In cases of tumors with distant lymph node metastasis, multiple lung lesions are often diagnosed as intrapulmonary metastasis. In contrast, if individual tumors constituting multiple lung cancers are pathologically different from each other in terms of histological type and/or cellular atypism, synchronous onset of multiple primary cancers is deemed likely. However, there are no specific clinical or radiologic features that can be used to confidently distinguish multiple primary cancers from intrapulmonary metastases in all cases, and the differential diagnosis is at times perplexing in the clinical setting. Their different biological activities may be responsible for prognostic differences, and patients with intrapulmonary metastasis tend to have a poor prognosis. Therefore, it is important to find ways to identify them by exploring new practical techniques and markers. Certain case studies have been published involving the diagnosis of multiple lung cancers based on the analysis of specific mutations such as *TP53*, *KRAS* and *EGFR* [1–6]. However, the analysis used in these studies does not cover all lung cancer-related gene mutations, so this approach obviously does not always allow for precise judgment. There may also be cases in which such an approach is difficult to apply clinically. As both a more precise and clinically applicable method, we undertook comprehensive mutation analysis with targeted deep sequencing and evaluated the possibility of identifying the clonality of individual lung cancers by using their mutations as a diagnostic marker. Furthermore, we evaluated the efficacy and broad clinical utility of this novel method from the standpoint of understanding the pathology and selecting the most appropriate treatment.

## RESULTS

### Patient characteristics

The 12 patients were divided into groups by the following characteristics (Table 1): 10 males, 2 female; 2 current smokers, 8 former smokers, 2 never smoker; pathological stage IA (4), IB (5), IIB (2) and IV (1). The patients’ age ranged between 54 and 82 (mean ± SD 70.6 ± 7.9) years. The maximum diameter of the tumors ranged between 8 and 60 (mean ± SD: 22.8 ± 12.7) millimeters.

**Table 1:** Patient characteristics

	Age	Gender	Smoking	Tumor location	Operative procedure	Size (mm)	pTNM	p-stage	Histopathology
1	57	male	former	rS6, rS2	middle and lower lobectomy, wedge resection	60, 15	T2bN1M0	IIB	① ker Sq, ly1, v1, pl1, ② ker Sq, ly0, v1, pl2
2	67	male	former	rS1, rS6	right upper lobectomy, wedge resection	20, 15	T2aN0M1a	IV	① inv Ad, lepid, ly0, v0, pl0, ② inv Ad, solid, ly0, v0, pl1
3	74	male	current	rS2, rS3	middle and upper lobectomy	35, 35	T2aN0M0	IB	① inv Ad, solid, ly1, v0, pl3, ② inv Ad, papillary, ly0, v1, pl0
4	82	male	former	rS10, rS6	right lower lobectomy	25, 20	T1bN0M0	IA	① inv Ad, lepid, ly0, v0, pl0, ② inv Ad, lepid, ly0, v0, pl0
5	54	male	former	rS3, leftS1+2	right upper lobectomy, left upper division segmentectomy	32, 17	T2aN0M0	IB	① inv Ad, lepid, ly1, v1, pl0, ② MIA, ly0, v0, pl0
6	73	female	never	rS10, rS4	partial resections	11, 8	T1aN0M0	IA	① inv muc Ad, ly0, v0, pl0 ② MIA, ly0, v0, pl0
7	72	male	former	leftS1+2	left upper division segmentectomy	15, 10	T1aN0M0	IA	① AIS, ly0, v0, pl0, ② MIA, ly0, v0, pl0
8	73	male	current	rS2, leftS8	right wedge resection, left lower lobectomy	20, 18	T2aN0M0	IB	① inv Ad, papillary, ly0, v0, pl0, ② small, ly0, v1, pl2
9	67	female	never	rS3, leftS9	right upper lobectomy, left lower lobectomy	20, 25	T2aN0M0	IB	① inv Ad, lepid, ly0, v0, pl0, ② inv Ad, papillary, ly0, v0, pl1
10	73	male	former	leftS3, leftS8	left pneumonectomy	33, 8	T2aN0M0	IB	① ker Sq, ly0, v1, pl0, ② AIS, ly0, v0, pl0
11	77	male	former	leftS6, leftS10	left lower lobectomy	52, 20	T2bN1M0	IIB	① ker Sq, ly1, v1, pl1, ② small, ly0, v1, pl2
12	78	male	former	rS2, rS3	right upper lobectomy	17, 15	T1aN0M0	IA	① MIA, ly0, v0, pl0, ② inv Ad, acinar, ly0, v0, pl0

S, segment; ker Sq, keratinizing squamous cell carcinoma; Ad, adenocarcinoma; inv, invasive; muc, mucinous; MIA, microinvasive adenocarcinoma; AIS, adenocarcinoma *in situ*.

### Targeted sequencing identified somatic mutations in lung cancers

**Figure 1:**
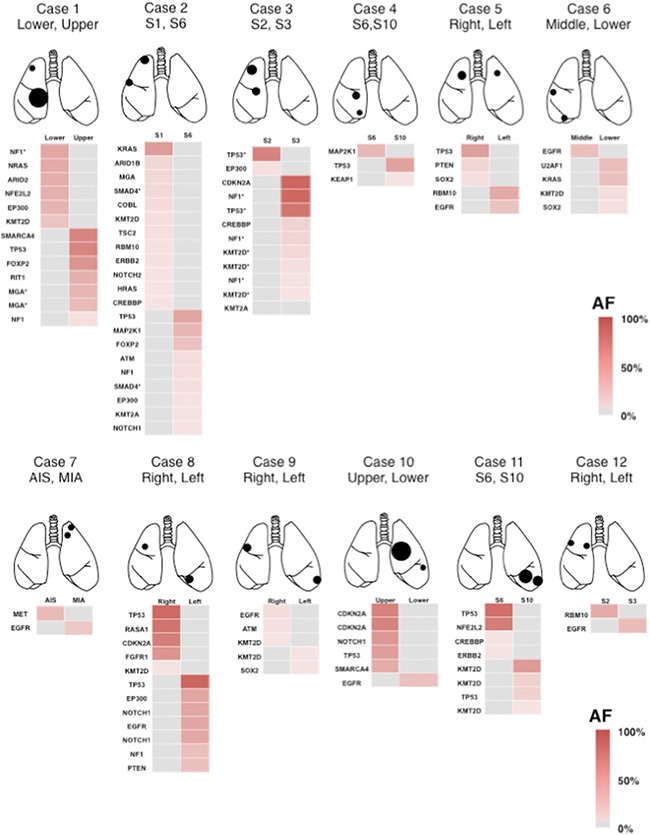
Heat map of the gene mutations in 12 patients with multiple lung cancers This map visualizes the gene mutations of each cancer, including those with an allele fraction below 20%. Two lung cancers in each patient were characterized by different gene mutations, and all of the cases were judged as having double primary lung cancers. Asterisk (*) indicates the different mutations in the same gene. S, segment; AIS, adenocarcinoma *in situ*; MIA, microinvasive adenocarcinoma; AF allele fraction.

We examined 24 surgically resected tumors from 12 patients for targeted sequencing, with their buffy coat samples utilized as normal controls. The mean coverage depth was 1063-fold for tumor samples (range: 273–2471) and 1192-fold for buffy coat samples (range: 472–2428). Sequence analyses identified 102 somatic mutations with an allele fraction ≥1% from 24 tumors (1–12 mutations per tumor) (Figure 1). Among these, 57 mutations (56%) were present at an allele fraction ≥20% (Table 2). In each patient, the gene, amino-acid substitution and nucleotide changes of these somatic mutations within the individual tumors constituting the multiple lung cancers lacked consistency (Table 2 and Figure 1). Thus, there was no overlap of mutations among the individual lung cancers detected in any patient. This finding demonstrated that the multiple lung cancers in each case were synchronously developing primary lung cancers (Table 2 and Figure 1). Three representative cases are presented below in detail.

**Table 2:** Mutation analysis of the multiple lung cancers

Case	Tumor location	Histology	Gene	Mutation	Position	Ref.	Tumor var.	AF
1			NF1	p.Tyr2476Phe	chr17:29677306	A	T	50%
			NRAS	p.Gln61Lys	chr1:115256528	G	T	49%
	right S6	SCC	ARID2	p.Thr219Ser	chr12:46215221	C	G	45%
			NFE2L2	p.Asp178His	chr2:178097182	C	G	44%
			EP300	p.Ser2328fs	chr22:41574692	GTCCT	GTCCCT	41%
			KMT2D	p.Arg2830Ter	chr12:49432651	G	A	29%
			SMARCA4	p.Glu371Ter	chr19:11098593	G	T	75%
			TP53	p.Arg248Trp	chr17:7577538	G	A	74%
	right S2	SCC	FOXP2	p.Pro277Leu	chr7:114271740	C	T	64%
			RIT1	p.Thr70Ser	chr1:155880247	T	A	43%
			MGA	p.Asp339His	chr15:41962107	G	C	39%
			MGA	p.Glu1249Lys	chr15:42021449	G	A	35%
2	right S1	Adeno	KRAS	p.Gly12Ala	chr12:25398284	C	G	58%
			TP53	p.Cys238Phe	chr17:7577108	C	A	51%
	right S6	Adeno	MAP23K1	p.Lys57Asn	chr15:66727455	G	T	39%
			FOXP2	p.Gln250Lys	chr7:114271658	C	A	31%
3			NF1	p.Glu2358fs	chr17:29670037	AAGTAT	A	95%
	right S3	Adeno	CDKN2A	splicesite_3	chr9:21970900	C	T	95%
			TP53	splicesite_5	chr17:7578556	T	C	85%
			TP53	splicesite_5	chr17:7578556	T	C	88%
	**left S1+2, bx**	**Adeno**	**NF1**	**p.Glu2358fs**	**chr17:29670037**	**AAGTAT**	**A**	**88%**
			**CDKN2A**	**splicesite_3**	**chr9:21970900**	**C**	**T**	**83%**
4	right S6	Adeno	MAP2K1	p.Ser222Thr	chr15:66774188	T	A	37%
	right S10	Adeno	TP53	p.Val173Glu	chr17:7578368	A	T	54%
5	left S1+2	Adeno	RBM10	p.Glu721Ter	chrX:47044469	G	T	48%
			EGFR	p.Leu858Arg	chr7:55259514	T	G	30%
	right S1	Adeno	TP53	p.Arg280Thr	chr17:7577094	C	G	56%
6	right S10	Adeno	U2AF1	p.Ser34Phe	chr21:44524456	G	G/A	33%
			KRAS	p.Gly12Asp	chr12:25398280	GCCAC	GCCAC/GCCAT	32%
	right S4	Adeno	EGFR	p.E746-R748 del	chr7:55242478	G	G/C	31%
7	left S1+2, AIS	Adeno	MET	p.Asp1028His	chr7:116412043	G	G/C	36%
	left S1+2, MIA	Adeno	EGFR	p.E746-A750 del	chr7:55242465	AGGAATTAAGAGAAGC	AGGAATTAAGAGAAGC/A	25%
8		Adeno	TP53	p.Arg249Ser	chr17:7577528	GATGGGCCTCCGGTTC	GATGGGCCTCCGGTTC/GATGGGACTCCGGTTC	84%
		Adeno	RASA1	p.Gly434Ter	chr5:86649020	G	G/T	78%
right S2	right S2	Adeno	CDKN2A	p.Asp74Tyr	chr9:21971138	C	C/A	72%
		Adeno	FGFR1	p.Lys436Glu	chr8:38277122	T	T/C	58%
		Adeno	KMT2D	p.Leu4467His	chr12:49425088	A	A/T	4%
		small	TP53	p.Cys242Phe	chr17:7577553	ATGCAGGAACTGT	ATGCAGGAACTGT/ATGAAG GAACTGT	86%
		small	EP300	p.Lys1783Arg	chr22:41573063	A	A/G	48%
		small	NOTCH1	p.Glu286Ter	chr9:139413904	C	C/A	47%
	left S8	small	EGFR	p.Phe481Leu	chr7:55227976	T	T/A	46%
		small	NOTCH1	p.Gln58Ter	chr9:139418400	G	G/A	45%
		small	NF1	p.Arg440Gln	chr17:29533316	G	G/A	30%
		small	PTEN	p.Thr277Ile	chr10:89720679	C	C/T	29%
9		Adeno	EGFR	p.Leu858Arg	chr7:55259515	TG	TG/GG	10%
	right S3	Adeno	ATM	p.Glu1971Lys	chr11:108181035	G	G/A	7%
		Adeno	KMT2D	p.Asp1749Glu	chr12:49437723	A	A/C	4%
	left S9	Adeno	SOX2	p.Glu282Val	chr3:181430993	A	A/T	4%
		Adeno	KMT2D	p.Asp632Glu	chr12:49445570	G	G/C	4%
10		SCC	CDKN2A	p.Asp108Tyr	chr9:21971036	C	C/A	69%
		SCC	CDKN2A	p.Leu104fs	chr9:21971041	ACGTCCAGCCGCGCC	ACGTCCAGCCGCGCC/A	69%
	left S3	SCC	NOTCH1	p.Cys1490Trp	chr9:139399878	G	G/C	56%
		SCC	TP53	p.Arg280Ile	chr17:7577094	GGTCTCT	GGTCTCT/GGTCTAT	52%
		SCC	SMARCA4	p.Asp779Tyr	chr19:11123685	G	G/T	43%
	left S8	Adeno	EGFR	p.Leu858Arg	chr7:55259515	TG	TG/GG	29%
11		SCC	TP53	p.Arg280Thr	chr17:7577094	GGTCTCT	GGTCTCT/GGTCTGT	80%
		SCC	NFE2L2	p.Leu30Phe	chr2:178098957	G	G/A	72%
	left S6	SCC	FGFR3	p.Ala636Thr	chr4:1807841	G	G/A	11%
		SCC	ERBB2	p.Asp1144His	chr17:37883959	G	G/C	4%
		SCC	CREBBP	p.Leu551Ile	chr16:3831230	G	G/T	4%
		small	KMT2D	p.Gln3969Leu	chr12:49426582	T	T/A	54%
	left S10	small	TP53	p.Val197Met	chr17:7578256	TCCACTCGGATAAGATGCTGAGGAGGGG	TCCACTCGGATAAGATGCTG AGGAGGGG/TCCATTCGGATAAGATGCTGAGGAGGGG	18%
		small	KMT2D	p.Cys778fs	chr12:49445134	A	A/AC	18%
		small	KMT2D	p.Asp632Glu	chr12:49445570	G	G/C	5%
12	right S2	Adeno	RBM10	p.Tyr573Ter	chrX:47040994	C	C/G	43%
	right S3	Adeno	EGFR	p.Leu858Arg	chr7:55259515	TG	TG/GG	33%

The entries in bold letters in Case 3 indicate the results from mutation analysis of bronchoscopic specimens, not surgically resected specimens. AF, allele fraction; var., variance; S, segment; SCC, squamous cell carcinoma; Adeno, adenocarcinoma; small, small cell carcinoma; bx, biopsy; chr, chromosome; AIS, adenocarcinoma in situ; MIA, microinvasive adenocarcinoma.

### Case presentations

**Figure 2:**
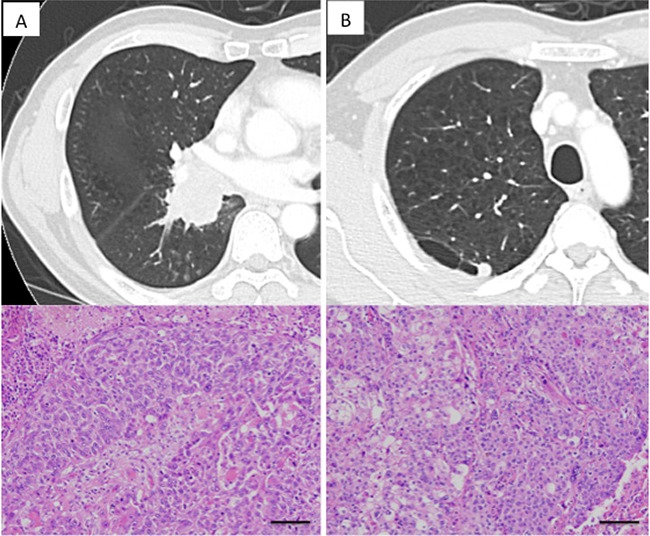
Radiologic findings of lung cancers in Case 1 (**A**) A large tumor affecting both the middle and lower lobes, diagnosed as squamous cell carcinoma. (**B**) A small nodular shadow neighboring the bulla was noted in the right upper lobe. Postoperative histological examination revealed it to be squamous cell carcinoma and distinction between primary and metastatic was difficult. Each scale bar indicates 100 μm.

Case 1: A 57 year-old man came to our department after abnormalities were detected on a chest radiograph taken in the course of a health checkup. A chest computed tomography (CT) revealed a tumor (60 mm in diameter) in the right middle and lower lobes (Figure 2A). Bronchoscopy yielded a diagnosis of squamous cell carcinoma. Preoperative CT and positron emission tomography (PET) showed a small nodule in the right upper lobe, which was interpreted by radiologists as intrapulmonary tumor metastasis (Figure 2B). Based on the assumption that the small nodule in the right upper lobe might be primary, surgery was performed. Right upper lobe wedge resection was performed and intraoperative pathological examination yielded a diagnosis of keratinizing squamous cell carcinoma. However, pathological distinction between the primary and metastatic tumors was deemed difficult, so the operation continued and middle and lower lobectomy was performed. The postoperative pathological examination rated both the upper lobe tumor and the middle/lower lobe tumor as keratinizing squamous cell carcinomas (Figure 2A and 2B), without any definitive judgment as to whether either tumor was primary or metastatic in nature. Upon targeted deep sequencing, the mutations in the upper lobe tumor involved *NF1*, *NRAS*, *ARID2*, *NFE2L2*, *EP300* and *KMT2D*, while the mutations in the middle/lower lobe tumor involved *SMARCA4*, *TP53*, *FOXP2*, *RIT1* and *MGA* (Table 2 and Figure 1). Thus, the mutation pattern differed completely between these two tumors, allowing for the determination of double primary lung cancers. Postoperative adjuvant chemotherapy was administered in this case. To date, the patient has had no recurrence for the 12 months that have elapsed since surgery.

**Figure 3:**
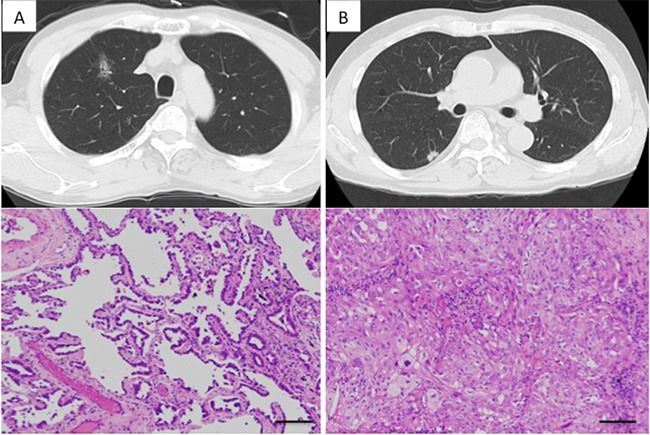
Radiologic findings of the lung cancers in Case 3 (**A**) Right upper lobe shadow: A shadow, primarily ground glass opacity and accompanied by partially solid consolidation. Histologically, it was diagnosed as lepidic predominant adenocarcinoma. (**B**) Right lower lobe shadow: Nodular shadow adjacent to the pleura. Histologically, it was diagnosed as solid predominant adenocarcinoma. Each scale bar indicates 100 μm.

Case 2: A 67 year-old man came to our department after a chest X-ray revealed an abnormal shadow. Chest CT showed ground-glass opacity (20 mm in diameter) in right S1 as well as a nodular shadow (15 mm in diameter) in right S6 (Figure 3A and 3B). Lung cancer was strongly suspected and the patient underwent surgery. When the chest was opened, a small volume of pleural effusion was observed, and adenocarcinoma cells were found by pleural lavage cytology. Thus, right upper lobectomy and partial resection of the right lower lobe were performed. Pathologically, the tumor in S1 was classified as a lepidic adenocarcinoma, pleural-invasion negative, and the tumor in S6 was classified as a solid adenocarcinoma, pleural-invasion positive. Because of slight differences in the histological findings of the two tumors (Figure 3A and 3B), double primary cancers were suspected. Upon targeted deep sequencing, significant mutations in the S1 tumor involved *KRAS*, while the significant mutations in the S6 tumor involved *TP53*, *MAP2K1* and *FOXP2* (Table 2 and Figure 1). Thus, the mutation profiles differed completely between the two tumors, supporting the view that these were double primary cancers. Postoperative chemotherapy was administered for this stage IV lung cancer. The patient has had no recurrence for 14 months to date since the surgery.

**Figure 4:**
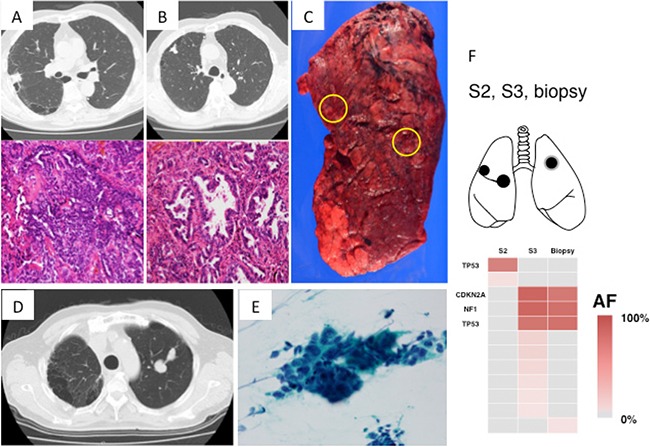
Radiologic, pathologic and genomic findings of the lung cancer in Case 3 (**A**) Lung cancer in right S2: solid predominant adenocarcinoma. (**B**) Lung cancer in right S3: papillary predominant adenocarcinoma. (**C**) Macroscopic view of the S2 and S3 lung cancers. Yellow circle indicates the locations of the cancers. (**D**) A solitary lung lesion newly developing in the left lung postoperatively. Radiologically, distinguishing between primary or metastatic cancer was difficult. (**E**) Histopathology of the bronchoscopic biopsy specimen: poorly differentiated adenocarcinoma. (**F**) Heat map of the gene mutations of 3 lung tumors. Mutation differed between right S2 and S3 tumors, but the significant mutations in right S3 tumor were homologous to those in left S1+2 tumor. S, segment; AF allele fraction.

**Figure 5:**
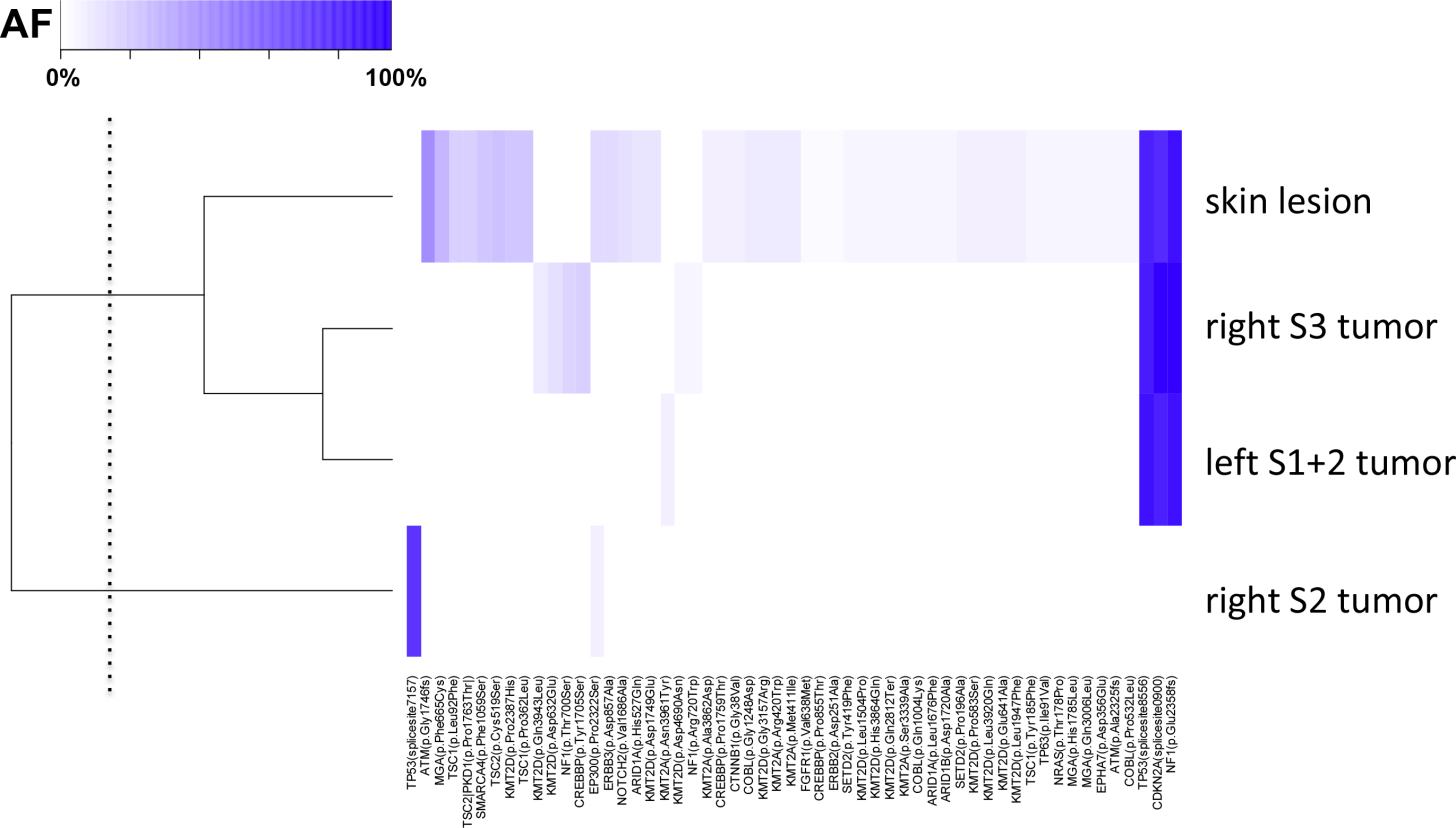
Cluster analysis of the point mutations in Case 3 The mutation data were standardized and presented as a heat map. The skin lesion harbored the common driver mutations with right S3 and left S1+2 cancers. By the cluster analysis, the lesions that share a similar pattern of allele fraction were identified. Unsupervised hierarchial clustering was used to group correlated lesions, with the dendrogram threshold level to establish two clusters indicated on the y-axis (dotted line). Column at bottom of heatmap shows the mutated genes and amino acid changes.

Case 3: A 74-year-old man visited our department for further examination of abnormalities on a routine chest radiograph. Chest CT revealed two tumors (35 mm in diameter) in the right upper lobe (Figure 4A and 4B). Bronchoscopy indicated adenocarcinoma. Right upper lobectomy was performed based on the diagnosis of double primary lung cancers (Figure 4C). Pathologically, the segment 2 (S2) tumor was rated as a solid adenocarcinoma and the S3 tumor as a papillary adenocarcinoma (Figure 4A and 4B), thus suggesting that this was likely a case of double primary cancers. On targeted deep sequencing, mutations in the S2 tumor involved *TP53* (chr17: 7577157, T→A splice site mutation), while mutations in the S3 tumor involved *NF1*, *CDKN2A* and *TP53* (chr17: 7578556, T→C splice site mutation) (Table 2 and Figure 1). *TP53* mutations were seen in both segments, but they differed in nucleotide position and variance. Such a complete difference in mutation patterns supported the judgment of double primary cancers. During outpatient follow-up, a new tumor developed in the left S1+2 at 6 months after surgery (Figure 4D). Bronchoscopy revealed it to be an adenocarcinoma (Figure 4E), but was unable to determine whether this new tumor was primary or metastatic. Therefore, targeted deep sequencing was performed with bronchoscopically biopsied specimens to compare the mutations with those found in the previously resected specimens. Upon performing this comparison, the mutation in the left S1+2 tumor involved *TP53*, *NF1* and *CDKN2A*, identical to that of the right S3 tumor (Figure 4F). Furthermore, the nucleotide position and variance of the mutation in S1+2 were entirely consistent with those in S3 (Table 2). Thus, a diagnosis of solitary, contralateral, intrapulmonary metastasis was made, and systemic chemotherapy was started. 10 months after the surgery, multiple intrapulmonary, liver and brain metastases developed and then a swelling skin lesion appeared on the left iliac region, which was biopsied to be a metastatic adenocarcinoma histologically. From the view of the mutation profiles, the predominant mutation in the skin lesion involved *TP53*, *NF1* and *CDKN2A*, identical to that of the right S3 adenocarcinoma, not S2 adenocarcinoma (Figure 5). According to the cluster analysis, the left lung tumor and the skin lesion cluster together genomically with the right S3 cancer and those clusters segregate away from the right S2 cancer (Figure 5). 16 months after the surgery, the patient died of his cancer due to respiratory failure.

## DISCUSSION

In cases of multiple lung cancers, clinical distinction between primary and metastatic tumors is sometimes difficult, making treatment selection challenging. Thus, we conducted lung cancer mutation analysis by targeted deep sequencing and found that mutations in individual lung cancers could serve as a clonal marker, allowing distinction of the clonality of individual tumors.

In Case 1, pre-operative distinction between primary and metastatic tumors on the basis of clinical course, diagnostic imaging, and pathological examination was difficult. Even experienced radiologists interpreted the PET and CT findings to indicate intrapulmonary metastasis, although the true diagnosis was double primary cancers. Thus, it is possible that certain multiple primary lung cancers are misdiagnosed as metastatic cancer based on pathology and radiology [7]. Our novel approach may help resolve the current dilemma of misdiagnosis in the clinical setting.

In Case 3, the mutation found in the right S3 tumor was consistent with that in the left S1+2 tumor, demonstrating that this was a case of lung cancers from the same clone (i.e., intrapulmonary metastasis). Consistency in multiple mutations, with complete consistency observed even in the position and patterns of base-pair substitutions, cannot be an accidental phenomenon. Although inconsistency between the two tumors was noted in mutations of less than 10% of the allele fraction, this can be explained by tumor heterogeneity. In general, cancers are composed of populations of cells with distinct molecular and phenotypic features, a phenomenon termed intratumor heterogeneity [8, 9]. Intratumor heterogeneity, associated with heterogeneous protein function, may foster tumor adaptation, phenotypic aggravation, and/or therapeutic failure through Darwinian selection [10, 11]. In contrast, a pivotal driver mutation serves as the trigger of clonal expansion and is estimated to be retained homogenously within the tumors of the same clone [11–14], regardless of whether they are intra- or extrapulmonary lesions, such as the skin metastasis in Case 3. These events can be explained by the “trunk and branch” mutation models, i.e., early somatic events that drive tumor growth or maintenance in early clonal progenitors are represented within the “trunk” of the tumor [8, 9]. Such trunk somatic aberrations, present at the early stages of tumor development, are likely to be ubiquitous events occurring at all sites of disease. In contrast, later somatic events that occur following branched separation of subclones represent heterogeneous events. Such subclonal heterogeneity may be spatially separated between regions of the same tumor or its metastatic sites [8–10]. In this context, clonally dominant “trunk” somatic aberrations are important clonal markers. Primary and metastatic tumors can be discriminated by determining whether such ubiquitous mutations are identical.

It can be relatively easy to diagnose multicentric primary lung cancers when their histological types are different. However, if they are of the same histological type, it is often difficult to discriminate multiple primary lung cancers from intrapulmonary metastasis. Particularly in cases such as Case 1 (multiple tumors classified as squamous cell carcinoma), making a distinction by pathology alone is difficult. A bronchoscopic examination involves a biopsy of part of the tumor, preventing assessment of the full pathological profile and occasionally making it difficult to distinguish between primary and metastatic tumors. In our cases, the bronchoscopically biopsied specimens were very small (about 1 mm^3^ each), but mutation analysis was possible, allowing comparative analysis of the mutations. Even when morphological and immunohistological features are nonhomogeneous among different parts of the tumors, the driver mutation serving as the trunk is prevalently retained within the tumors of the same clone [11–14]. Therefore, distinction of clonality based on mutation analysis is suggested to be more specific and definitive than histological distinction. Furthermore, based on the hierarchical clustering results, we could subclassify the four lesions of clonal diversity into two distinct groups. Thus, cluster analysis is helpful for evaluating the clonal progression of the cancer among the multiclonal cancer lesions. Even if part of the mutation profile is matched between two tumors to some degree, these tumors can be presumed to be primary or metastatic by performing cluster analysis to estimate the genetic distance between them.

Detterbeck et al. summarized clinical and pathologic criteria to distinguish second primary versus metastatic tumors; they concluded that few features are definitive and that it is difficult to define criteria that conclusively establish that the tumors are identical; finding similarities is not sufficient [15, 16]. In our method, when clonally dominant mutations with high allele fractions are also completely identical at the base-pair level, they are clearly defined as the same clone, as we showed in the presentation of Case 3. In other words, comparison of “trunk” mutations could yield definitive criteria. If “trunk” mutations are homologous, it would be easy to determine whether two tumors are the same.

In mutation analysis, many previous studies assessed particular mutations to define clonality, assuming that a match of a few (one to five) markers defines a single clone whereas a difference defines separate cancers [17–21]. However, although one to five mutations that are not “trunk” mutations are compared, their differences are not significant as the basis for discriminating between primary and metastatic tumors, and this characterization is associated with potential misclassification. According to our method, comprehensive mutation analysis is first performed to identify “trunk” mutations of each cancer, which are then compared to define their clonality. These criteria are not so much suggestive as definitive and reliable. In addition, they are relatively simple because they are measurable by next-generation sequencing alone. Moreover, the decision criteria are generally clear and intuitive. Thus, we expect that our method will be widely adopted as a standard diagnostic method in daily clinical practice in the future.

When treatment methods are selected for multiple lung cancers, it is necessary to consider which of the multiple cancers will affect the prognosis of patients most. In Case 2, malignant pleural effusion was noted, and the disease was diagnosed as stage IV. The factor responsible for progression to such an advanced stage was identified as the S6 adenocarcinoma with pleural invasion between the two adenocarcinomas. Therefore, treatment for this case will target the S6 adenocarcinoma. At present, molecular-targeted therapies of lung cancer are confined to those targeting *EGFR* and *ALK* mutations. The development of novel molecular-targeted therapies would enable postoperative adjuvant chemotherapy and recurrent tumor treatment tailored to the features of mutations in individual cancers. Thus, in cases of multiple lung cancers, checking mutations is also important based on selection of the most effective medical treatments.

In conclusion, when dealing with synchronous or metachronous multiple lung cancers, checking the differences in the mutation profile among multiple tumors will clarify the clonal origin of the tumors and enable distinction between primary and metastatic tumors with high specificity, even in cases where pathological distinction is not possible. Thus, treatment tailored to the features of individual cases will be possible. Furthermore, if mutation analysis is performed for bronchoscopically collected specimens, pre-operative diagnosis will be possible, and the treatment strategy for multiple lung cancers may thus be devised rationally.

## MATERIALS AND METHODS

### Patients and sample preparation

The study involved 12 patients who underwent surgery in our department for synchronous multiple lung cancers between July 2014 and March 2016. These patients provided written informed consent for the genetic research studies, which were performed in accordance with protocols approved by the Institutional Review Board at our hospital.

The serial section of formalin-fixed, paraffin-embedded (FFPE) tissue was stained with hematoxylin-eosin and then micro-dissected using an ArcturusXT laser-capture microdissection system (Thermo Fisher Scientific, Tokyo, Japan). DNA was extracted using the QIAamp DNA FFPE Tissue Kit (Qiagen). FFPE DNA quality was checked using primers for ribonuclease P (RNase P) locus. A peripheral blood sample was drawn from each patient just prior to surgery. Buffy coat were isolated following centrifugation and DNA was extracted from buffy coat using the QIAamp DNA Blood Mini Kit (Qiagen, Tokyo, Japan).

### Targeted deep sequencing and data analysis

A panel targeting the exon of 53 lung cancer-associated genes (see Supplementary Table 1) was established to perform targeted sequencing. We searched the literature and selected these genes based on the following criteria: (a) genes often involved in lung cancer reported TCGA [22, 23] and other projects [24–28] or (b) genes frequently mutated in lung cancer from the COSMIC database (http://cancer.sanger.ac.uk/cancergenome/projects/cosmic). The primer design for the targeted sequencing was performed by Ion AmpliSeq designer software (Thermo Fisher Scientific), as we previously reported [29, 30]. Sequencing libraries were prepared using Ion AmpliSeq Library kit (Thermo Fisher Scientific), according to the manufacturer's instruction. After barcode ligation using Ion Xpress Barcode Adapters kit (Thermo Fisher Scientific), library samples were purified using Agencourt AMPure XP reagent (Beckman Coulter, Tokyo, Japan) and subsequently quantified using Ion Library Quantitation Kit (Thermo Fisher Scientific). The libraries were templated with the Ion PI Template OT2 200 Kit v3 (Thermo Fisher Scientific). Sequencing was carried out on an Ion Proton (Ion Torrent) using the Ion PI Sequencing 200 Kit v3.

The sequence data were processed using standard Ion Torrent Suite Software running on the Torrent Server. Raw signal data were analyzed using Torrent Suite version 4.0. The pipeline included signaling processing, base calling, quality score assignment, read alignment to the human genome 19 reference (hg19), quality control of mapping and coverage analysis. Following data analysis, annotation of single nucleotide variants, insertions and deletions was performed by the Ion Reporter Server System (Thermo Fisher Scientific), and lymphocytes from peripheral blood DNA were used as a control to detect any variants (Tumor-Normal pair analysis). Sequence data were visually confirmed with the Integrative Genomics Viewer.

Unsupervised hierarchical clustering was used to identify potential distinct subgroups among the multi-clonal lesions based on the genetic profiling. The genetic informations were standardized, clustered and visualized with the CLUSTER and TREEVIEW programs.

## SUPPLEMENTARY MATERIALS TABLES



## Abbreviations

PCR: polymerase chain reaction
CT: computed tomography
PET: positron emission tomography
S: segment
