# Supplementary material for: Mutational analysis of multiple lung cancers: Discrimination between primary and metastatic lung cancers by genomic profile
Source: Oncotarget. 2017 Mar 10;8(19):31133–43. doi: 10.18632/oncotarget.16096 (PMC5458195; doi:10.18632/oncotarget.16096)
Supplement: Supplementary file 1 [file oncotarget-08-31133-s001.pdf]

Mutational analysis of multiple lung cancers: Discrimination between primary and metastatic lung cancers by genomic profile

Supplementary Materials

Supplementary Table 1: The genes targeted in the cancer panel

No	Gene symbol	Chromosome	Number of Amplicons	Total Bases	Covered Bases	Overall Coverage
1	AKT1	chr14	26	1573	1497	95%
2	AKT2	chr19	27	1576	1543	98%
3	AKT3	chr1	30	1624	1624	100%
4	ARID1A	chr1	76	7058	6023	85%
5	ARID1B	chr6	75	6950	5965	86%
6	ARID2	chr12	71	5718	5643	99%
7	ASCL4	chr12	5	532	382	72%
8	ATM	chr11	147	9791	9439	96%
9	BRAF	chr7	37	2481	2224	90%
10	CDKN2A	chr9	9	962	612	64%
11	COBL	chr7	48	4151	3977	96%
12	CREBBP	chr16	96	7639	7071	93%
13	CTNNB1	chr3	32	2486	2486	100%
14	CUL3	chr2	42	2561	2495	97%
15	EGFR	chr7	60	4189	4135	99%
16	EP300	chr22	90	7555	7182	95%
17	EPHA7	chr6	44	3175	3154	99%
18	ERBB2	chr17	57	4080	3808	93%
19	ERBB3	chr12	59	4440	4374	99%
20	FGFR1	chr8	41	2825	2816	100%
21	FGFR2	chr10	43	2910	2842	98%
22	FGFR3	chr4	34	2752	2215	81%
23	FOXP2	chr7	36	2487	2469	99%
24	HRAS	chr11	11	683	683	100%
25	KEAP1	chr19	24	1925	1845	96%
26	KMT2D	chr12	192	17154	15854	92%
27	KRAS	chr12	10	737	681	92%
28	MAP2K1	chr15	18	1292	1239	96%
29	MET	chr7	59	4427	4396	99%
30	MGA	chr15	110	9428	9345	99%
31	MLL	chr11	144	12279	11875	97%
32	NF1	chr17	136	9161	9023	99%
33	NFE2L2	chr2	23	1868	1826	98%
34	NOTCH1	chr9	99	8008	7078	88%
35	NOTCH2	chr1	101	7809	7539	97%
36	NRAS	chr1	9	610	610	100%
37	PIK3CA	chr3	50	3407	3282	96%
38	PTEN	chr10	18	1302	1223	94%
39	RASA1	chr5	55	3412	3216	94%
40	RB1	chr13	55	3057	2902	95%
41	RBM10	chrX	48	3228	3079	95%
42	RIT1	chr1	13	771	771	100%
43	SETD2	chr3	91	7905	7663	97%
44	SLIT2	chr4	76	4972	4854	98%
45	SMAD4	chr18	24	1769	1715	97%
46	SMARCA4	chr19	74	5399	5055	94%
47	SOX2	chr3	9	964	883	92%
48	STK11	chr19	23	1392	1343	97%
49	TP53	chr17	22	1383	1351	98%
50	TP63	chr3	34	2360	2227	94%
51	TSC1	chr9	49	3705	3603	97%
52	TSC2	chr16	92	5834	5677	97%
53	U2AF1	chr21	15	880	870	99%
